# Alzheimer’s Disease Mortality Rate: Correlation with Socio-Economic and Environmental Factors

**DOI:** 10.3390/toxics12080586

**Published:** 2024-08-13

**Authors:** Valfran da Silva Lima, Yasmin Gabriele Ferreira, Júlio Cezar de Oliveira, Vanessa de Almeida Raia, Ludmila Barbosa Bandeira Rodrigues Emerick, Lucinéia Reuse Albiero, Valéria Dornelles Gindri Sinhorin, Guilherme Luz Emerick

**Affiliations:** 1 Instituto de Ciências da Saúde, Universidade Federal de Mato Grosso—ICS/CUS/UFMT, Campus Sinop, Avenida Alexandre Ferronato, 1200, Cidade Jardim, Sinop 78550-728, MT, Brazil; valfransl@hotmail.com (V.d.S.L.); yasminferreiray@gmail.com (Y.G.F.); biojborges@gmail.com (J.C.d.O.); vanessaraiaufrrj@gmail.com (V.d.A.R.); ludbbremerick@gmail.com (L.B.B.R.E.); lucineia_albiero@hotmail.com (L.R.A.); 2 Programa de Pós-Graduação em Ciências em Saúde, Instituto de Ciências da Saúde, Universidade Federal de Mato Grosso—ICS/CUS/UFMT, Sinop 78550-728, MT, Brazil; 3 Instituto de Ciências Exatas e da Terra, Universidade Federal de Mato Grosso—ICET//UFMT, Cuiabá 78060-900, MT, Brazil; valeria.sinhorin@ufmt.br

**Keywords:** mortality rate, Alzheimer’s disease, life expectancy at birth, Human Development Index, pesticides

## Abstract

The progressive increase in the number of deaths caused by Alzheimer’s disease (AD) in Brazil and around the world between 2010 and 2020 raises questions in scientific society. At the same time, there is also an increase in life expectancy at birth (LEB). Thus, the aim of this study was, for the first time, to compare the increase in AD mortality rate (ADMR) in Brazilian regions over the years 2010 to 2020 with the increase in LEB, and investigate the possible correlation between these demographic transition phenomena and pesticide sales and exposure during this period. Data were extracted from the Brazilian Institute of Geography and Statistics (IBGE), from the Department of Informatics and Technology of the Brazilian Ministry of Health (DATASUS) and from the Brazilian Institute of the Environment and Renewable Natural Resources (IBAMA). There was a significant increase in life expectancy at birth and in ADMR over the years between 2010 and 2020 in all Brazilian regions, with the female population in the South region being the most affected. In conclusion, there is a strong positive correlation between the increase in ADMR and LEB; ADMR and Human Development Index (HDI) and ADMR and pesticide sales and exposure in Brazil over the years studied.

## 1. Introduction

Future projections for the world population predict that the elderly population over the age of 65 will reach 1 billion by 2030, equivalent to 12% of the global population. These changes in demographic characteristics towards the increase in the elderly population present not only a socioeconomic setback, but also a major challenge for public health, as it is closely associated with the increased prevalence of age-related diseases, such as dementia [1,2].

Brazil is experiencing a phenomenon of demographic transition that results in changes in the size of various age groups of the population. In 1970, there was an age pyramid with a wide base and a narrow top. In 2020, there are three older age groups, reducing the proportion of children and young people and increasing the number of adults and elderly people. This becomes clear when looking at the age pyramid projected for 2060, when the age structure of the population will have a rectangular shape and the expectation is that the percentage of elderly people will exceed the number of children [3].

Life expectancy at birth (LEB) is an important indicator for measuring socioeconomic development, as its increase reflects an improvement in the living conditions of a given population [4]. According to the demographic census carried out by the Brazilian Institute of Geography and Statistics (IBGE), Brazil achieved a significant increase in life expectancy at birth, passing from 73.5 years in 2010 to 76.8 years in 2020 [5]. LEB expresses the longevity of the population and can be calculated by taking the number corresponding to an initial generation of births (l0) and the cumulative time lived by that same generation until the limit age (T0). It is necessary to obtain the quotient T0/l0 to obtain LEB, which may vary according to sex, race and other demographic factors [6]. In this context, another important concept is the Human Development Index (HDI) that estimates the development stage of populations, based on access to knowledge, a long and healthy life and a decent standard of living. This indicator varies from zero to one and is applied all around the world [7].

The most common form of neurodegenerative dementia in old age is Alzheimer’s disease (AD). Although its exact pathogenesis is not yet well understood, the interaction of genetic and environmental factors may be involved in the initiation of pathogenesis. One of the factors attributed as a probable cause of neurodegeneration is the accumulation of misfolded proteins, and very recently, the hypothesis of an involvement of the immune system in the pathogenesis of AD through neuroinflammation was raised, since peripheral immunological changes are associated with cognitive dysfunction [8,9].

Cellular senescence through the loss of normal functions and regeneration capacity contributes significantly to the pathophysiology of aging and age-related diseases including AD [10]. Epidemiological studies estimate that life expectancy after the onset of symptoms of AD varies between 3 and 12 years. Among the main factors that are associated with mortality include advanced age, male sex and comorbidities. The biggest risk factor for late-onset AD is aging, accounting for more than 95% of cases [2].

However, there are several additional factors that may increase the risk for the development of AD, including environmental influences such as the exposure to pesticides of various classes [11]. Brazil has been considered one of the largest consumers of pesticides in the world, with estimated sales for 2020 at more than 685,000 tons [12]. In the period from 2007 to 2017, in Brazil, around of 40,000 cases of acute intoxication induced by pesticides were registered, and chronic health effects of exposures at low doses are not included in these statistics, which may indicate even more alarming numbers [13].

Due to changes in population demographics and the impact of AD on public health, we hypothesize that LEB, HDI and pesticide sales (use) and exposure could be correlated to the deaths caused by AD in different regions of Brazil over the period between 2010 and 2020. Thus, the aim of this study is, for the first time, to compare the increase in AD mortality in Brazilian regions over the years from 2010 to 2020, with the increase in LEB and investigate the possible correlation between these demographic transition phenomena and pesticide sales during this period. This time frame (2010–2020) is chosen because it was the last decade pre-COVID-19 pandemic and because the Brazilian notification system presented reliable and traceable data. The intention is to analyze the changes in socio-economic and environmental factors that may influence the deaths caused by Alzheimer’s disease in Brazil. For future projections, we seek to understand how these factors will have changed after the inclusion of COVID-19 as a possible source of bias in the context of deaths caused by AD.

## 2. Materials and Methods

### 2.1. Study Design

**Figure 1:**
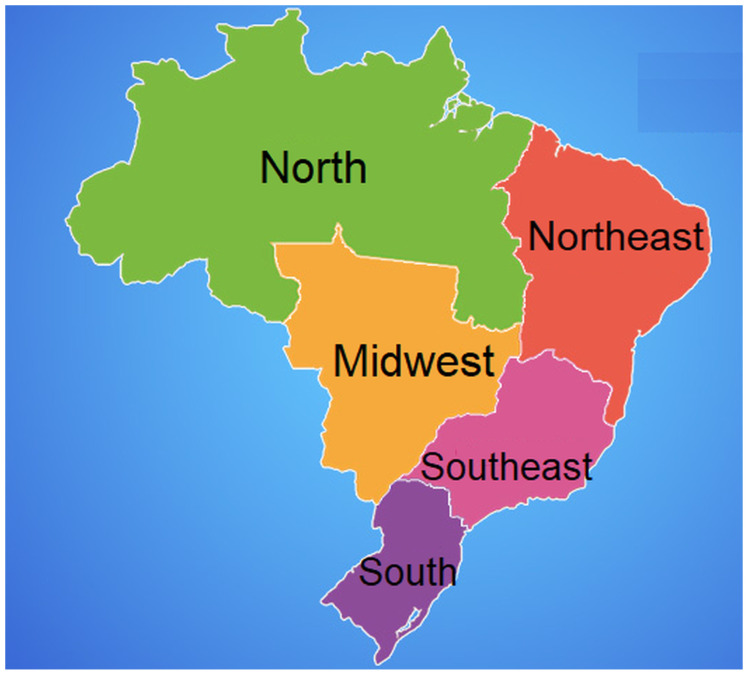
Map illustrating the five Brazilian regions in different colors. The map was extracted from the Brazilian Institute of Geography and Statistics [16].

This is an observational study characterized as ecological, descriptive and analytical with a quantitative approach, whose units of analysis were made considering the major Brazilian regions (Figure 1): South, Southeast, North, Northeast and Midwest, and the period analyzed was between the years 2010 and 2020 [14]. The indicators studied were LEB, AD mortality rate (ADMR), Human Development Index (HDI) and pesticide sales in Brazil. Ethical aspects were followed in accordance with the Resolution of the National Health Council n° 510/2016, whose research falls under item II, which uses publicly accessible information, and therefore does not require processing in the Research Ethics Committee system [15].

### 2.2. Life Expectancy at Birth (LEB)

LEB indicates an improvement in the quality of life and health conditions of the population in question and is not affected by the effects of the age structure of the population, as is the case with the crude mortality rate. It has been used in Brazil to support the planning, management and evaluation of health policies related to the rate of aging of the population. LEB can be calculated by the quotient of the cumulative time lived by the generation until the limit of age specified (T0) divided by the number corresponding to an initial generation of births (l0). For the present work, data with LEB information were extracted from the IBGE website through the following link: https://sidra.ibge.gov.br/tabela/7362#resultado, accessed on 9 July 2024 [17].

### 2.3. Human Development Index (HDI)

According to United Nations [7], HDI constitutes statistical data created to counter the purely economic data used to measure the wealth of countries and analyze development based on a long and healthy life, knowledge (adult literacy rate and education levels of the general population) and a decent standard of life (Gross Domestic Product divided by total population of the specific country). In Brazil, the official HDI is published by IBGE through the following website: https://cidades.ibge.gov.br/brasil/pesquisa/37/30255?ano=2010, accessed on 9 June 2024 [18].

### 2.4. Alzheimer Disease Mortality Rate (ADMR)

To analyze the number of deaths caused by AD, data were extracted from the Mortality Information System (SIM) through the link http://tabnet.datasus.gov.br/cgi/tabcgi.exe?sim/cnv/obt10uf.def (accessed on 9 June 2024). For the present work, the number of deaths caused by AD among Brazilian regions (Figure 1) between 2010 and 2020 was considered. To this end, data were separated into three categories: (1) female, (2) male and (3) both sexes. All data were collected secondarily through the Information Technology Department of the Brazilian Unified Health System (DATASUS) with the help of the TabNET^®^ software (version 2024), a free online government platform with public access [19].

### 2.5. Pesticide Data

Pesticide commercialization in Brazil is controlled by the Brazilian Institute of the Environment and Renewable Natural Resources (IBAMA), which is part of the Ministry of the Environment and Climate Change. The pesticide marketing reports that were used in this work are published annually and can be obtained through the following webpage: https://www.gov.br/ibama/pt-br/assuntos/quimicos-e-biologicos/agrotoxicos/relatorios-de-comercializacao-de-agrotoxicos, accessed on 21 July 2024 [20]. The number of cases of pesticide acute intoxication of each Brazilian region was obtained secondarily through the Information Technology Department of the Brazilian Unified Health System (DATASUS) using the following link: http://tabnet.datasus.gov.br/cgi/tabcgi.exe?sinannet/cnv/Intoxbr.def, accessed on 18 July 2024 [21].

### 2.6. Statistical Analysis

Data from the present work were tabulated using Microsoft Office Excel^®^ (version 365 for Windows). The Shapiro–Wilk test was used to confirm the normal distribution of all data. The ADMR of Brazilian regions (Midwest, Northeast, North, Southeast and South) was calculated using the number of deaths available in the system of DATASUS as the numerator and population data obtained from IBGE censuses of 2010 to 2020 as the denominator [19]. The result of this division was multiplied by 1,000,000 (one million) inhabitants for each region, according to Equation (1).

(1) $$ADMR=\left(\frac{Number\;of\;deaths\;caused\;by\;AD\;in\;the\;specific\;region}{Population\;of\;the\;specific\;region}\right)\times 1,000,000$$

Linear regression was calculated using the method of least squares and Pearson’s correlation was used to verify the causality of each variable analyzed (ADMR versus HDI, ADMR versus LEB, ADMR versus pesticide commercialization and number of cases of pesticide intoxication versus ADMR of each Brazilian region). Differences among the values of LEB and ADMR were examined for statistical significance by one-way ANOVA (analysis of variance) followed by Tukey’s test for multiple comparisons. GraphPad Prism Software (version 8.0 for Windows, San Diego, CA, USA) was used to perform all statistical analyses. Values of *p* < 0.05 were considered significant.

## 3. Results

### 3.1. LEB

**Figure 2:**
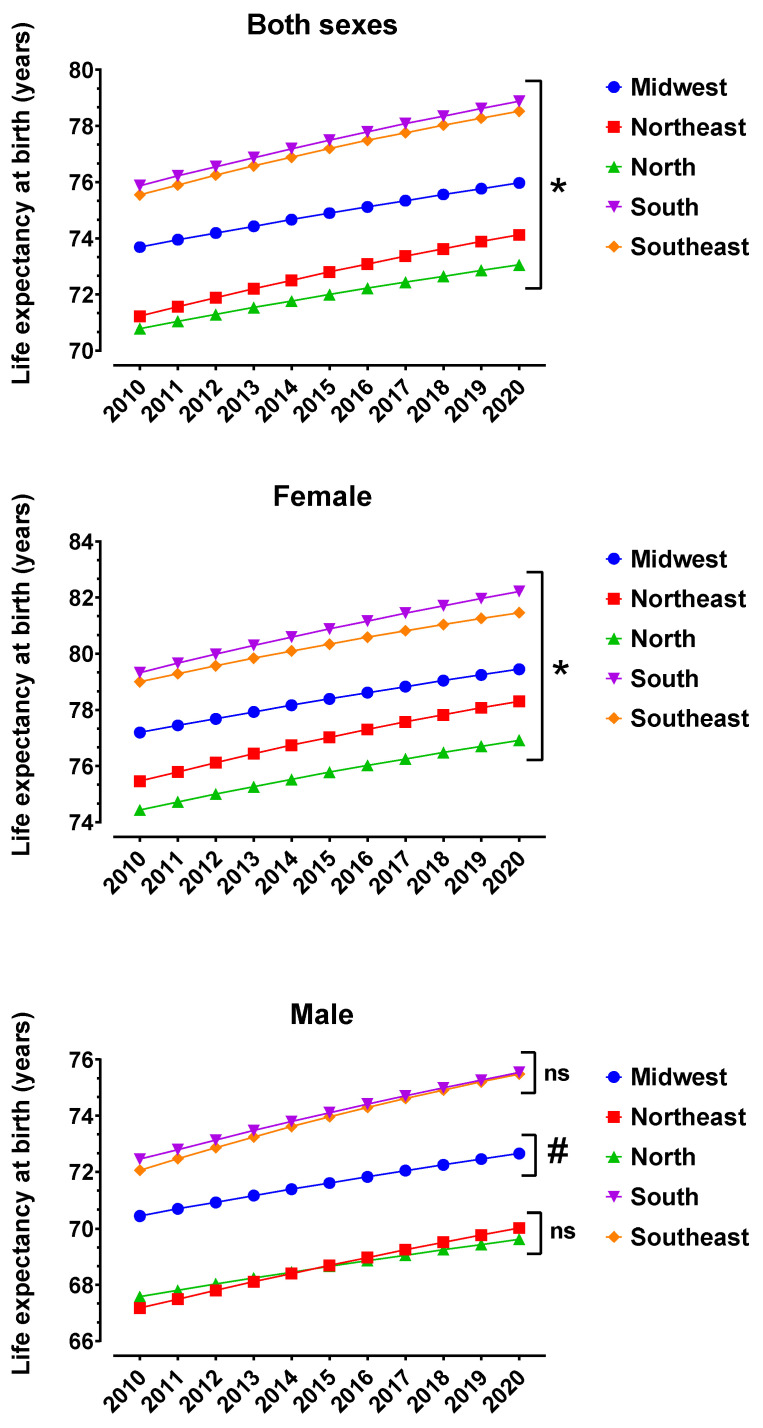
Life expectancy at birth in Brazilian regions between 2010 and 2020. Female, male and both sexes were evaluated separately to enable comparisons with the mortality rate. * means that all lines are statistically different from each other according to ANOVA followed by Tukey’s test for multiple comparisons. # means statistical difference from the other regions according to ANOVA followed by Tukey’s test for multiple comparisons. ns means not significant when comparing the two regions.

Figure 2 shows that the female population has a higher LEB than male, and all five regions of the country showed growth in LEB from 2010 to 2020, for male, female and both sexes. In relation to the years accumulated in LEB during the decade under study, the male population of the Southeast region and the female population of the South region had positive gains of 3.43 and 2.89 years, respectively. Figure 2 also shows that the South region presented higher LEB than the other four regions, with statistically significance for female and both sexes, but not for the male population when the South and Southeast regions were not statistically different.

### 3.2. ADMR

**Figure 3:**
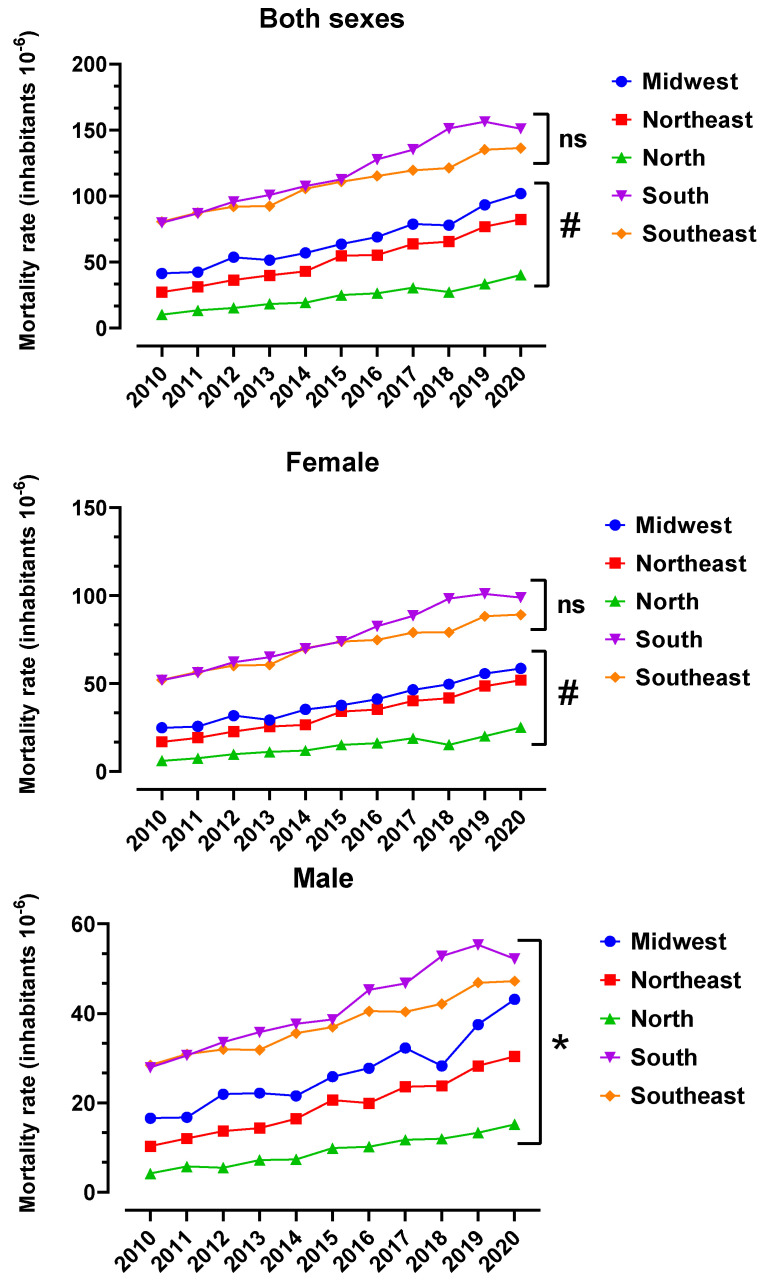
Alzheimer’s disease mortality rate (ADMR, 10^−6^ inhabitants) in Brazilian regions between 2010 and 2020. The ADMR of Brazilian regions (Midwest, Northeast, North, South and Southeast) was calculated using the number of deaths available in the system of DATASUS as the numerator and population data obtained from IBGE censuses of 2010 to 2020 as the denominator. The result of this division was multiplied by 1,000,000 (one million) inhabitants for each region, according to Equation (1), Section 2.6. * means that all the lines are statistically different from each other according to ANOVA followed by Tukey’s test for multiple comparisons. # means statistical difference from the other regions according to ANOVA followed by Tukey’s test for multiple comparisons. ns means not significant when comparing the two regions.

Figure 3 shows ADMR (10^−6^ inhabitants) over the years of 2010 to 2020 with a higher prevalence in females, corresponding to 63.06% of cases. When comparing the major Brazilian regions over the years 2010 to 2020, a growth pattern was observed, with an increase of approximately two times in both sexes (Figure 3). ADMR was calculated based on the total population of each region and, for both sexes, South (32.09%) and Southeast (29.43%) presented higher ADMR than the other regions, with the Midwest, Northeast and North regions contributing with 17.95%, 14.16% and 6.37% of deaths due to AD, respectively.

**Figure 4:**
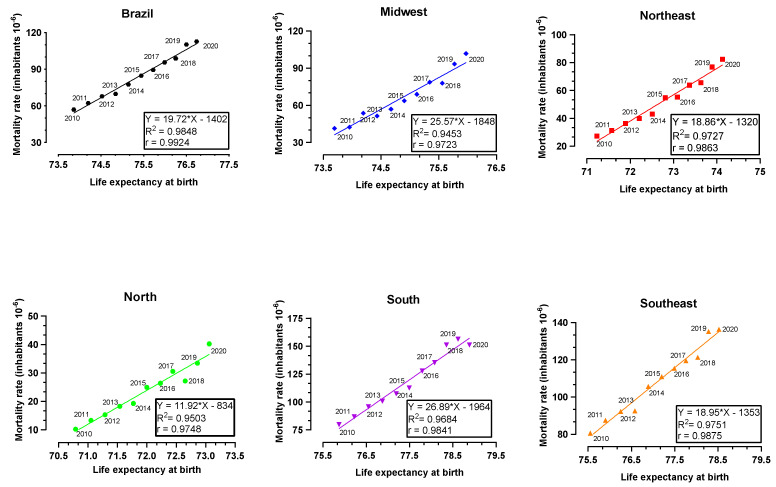
Alzheimer’s disease mortality rate (ADMR, 10^−6^ inhabitants) in Brazilian regions between 2010 and 2020 versus life expectancy at birth in Brazilian regions between 2010 and 2020. The equation (Y = a*X + b) of the curve was calculated using the method of least squares. Pearson’s correlation (r) was calculated for Brazil and each major region, and all were statistically significant (*p* < 0.0001).

Figure 4 shows Pearson’s correlation when comparing the variables LEB and ADMR, in each of the Brazilian regions over the years from 2010 to 2020. LEB correlated positively and significantly with ADMR in the North (r = 0.9748), Northeast (r = 0.9863), Southeast (r = 0.9875), South (r = 0.9841), Midwest (r = 0.9723) and the whole of Brazil (r = 0.9924). The R-squared values of the regression curves were all bigger than 0.94 and the equation was constructed for all regions and for Brazil as a whole.

### 3.3. HDI

**Figure 5:**
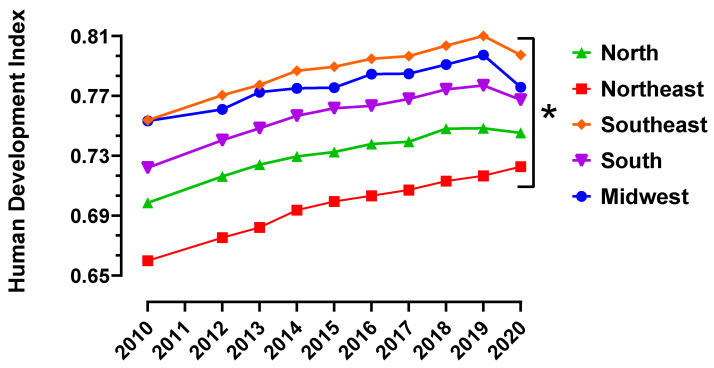
Human Development Index (HDI) by Brazilian major region calculated between the years 2010 and 2020. * means that all the lines are statistically different from each other according to ANOVA followed by Tukey’s test for multiple comparisons. In Brazil, the official HDI is published by IBGE through the following website: https://cidades.ibge.gov.br/brasil/pesquisa/37/30255?ano=2010 (accessed on 9 June 2024).

HDI was evaluated among the major Brazilian regions, as shown to Figure 5. The five regions presented HDI with statistical significance. The Southeast region presented an average HDI of 0.7880 for the period evaluated, followed by the Midwest (0.7771), South (0.7579), North (0.7320) and Northeast (0.6973). The Northeast region presented the greatest variation between the minimum HDI in 2010 (0.6598) and maximum in 2020 (0.7228) and the Midwest region presented the smallest variation between the minimum HDI in 2010 (0.7533) and maximum in 2019 (0.7973).

**Figure 6:**
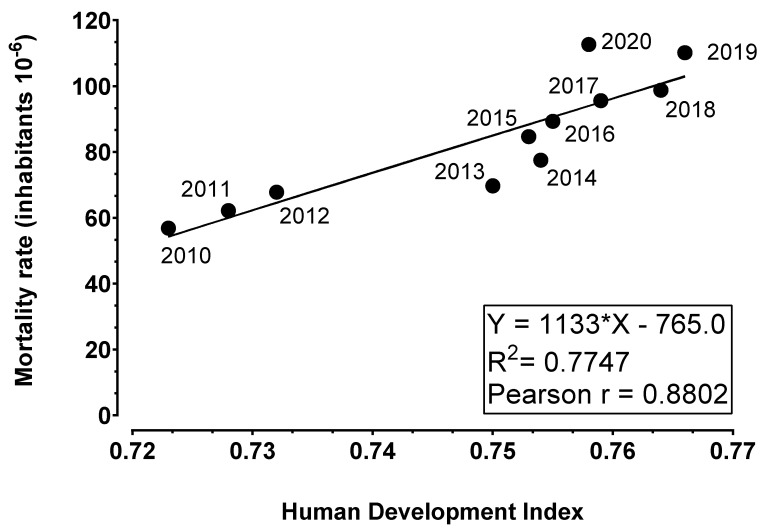
Alzheimer disease mortality rate (ADMR, 10^−6^ inhabitants) versus Human Development Index (HDI) of Brazil between the years 2010 and 2020. The equation (Y = a*X + b) of the curve was calculated using the method of least squares. A Pearson’s correlation (r) of 0.8802 was statistically significant (*p* = 0.0004).

Figure 6 correlates the ADMR and HDI of Brazil over the years from 2010 to 2020. Pearson’s coefficient r was 0.8802 and showed statistically significance with *p* = 0.0004. R-squared was also calculated using the method of least squares and was equal to 0.7747. The lowest HDI registered for the period evaluated was in 2010 with 0.7230 and the highest was registered in 2019 with 0.7660. On the other hand, the ADMR registered in this period was progressively greater, with the highest value of 112.65 achieved in 2020.

### 3.4. Pesticides

**Figure 7:**
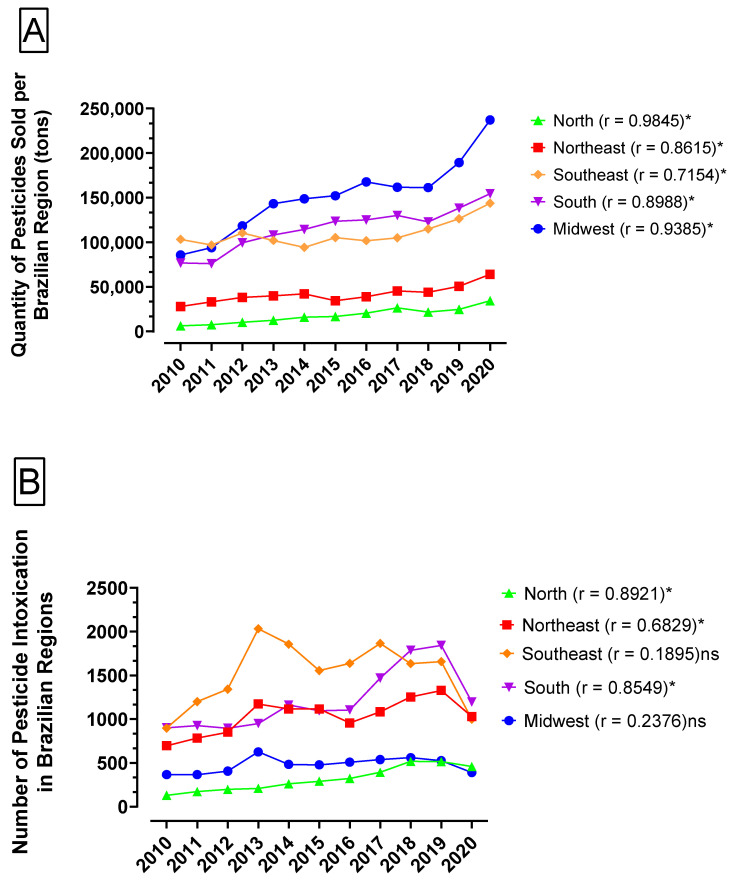
(**A**) Quantity of pesticides sold per Brazilian region over the years from 2010 to 2020 in tons of active principle. Pearson’s correlation (r) was calculated for each comparison between the quantity of pesticides sold per Brazilian region and the ADMR of the respective region. * means statistically significant (*p* < 0.05). (**B**) Number of pesticide intoxication cases registered in each Brazilian region over the years from 2010 to 2020. Pearson’s correlation (r) was calculated for each comparison between the number of pesticide intoxication cases registered in each Brazilian region and the ADMR of the respective region. * means statistically significant (*p* < 0.05). ns means not significant.

Figure 7A presents the quantity of pesticides sold per Brazilian region over the years from 2010 to 2020. Pearson’s coefficient (r) for each comparison opposing the quantity of pesticides sold per Brazilian region to the ADMR of each region was calculated and all five comparisons presented a correlation with statistical significance. The North region presented the highest coefficient (0.9845) and the Southeast region presented the lowest coefficient (0.7154). The quantity of pesticides sold in Brazil was progressively greater over the years from 2010 and 2020, being registered for 2010 at about 384,501 tons and 685,746 tons for 2020. Figure 7B shows the number of cases of acute intoxications caused by pesticides in Brazilian regions over the years from 2010 to 2020 and Pearson’s coefficient (r) for each comparison opposing the number of cases of pesticide intoxication to the ADMR of each region. The number of cases of registered intoxication was progressively greater over the years for most of the regions with 2020 showing a reduction when compared to the previous year. The North, Northeast and South regions presented a coefficient of correlation with statistical significance (*p* = 0.0002, 0.0206, and 0.0008, respectively). On the other hand, the Southeast and Midwest regions did not present a statistical correlation (*p* = 0.5767 and 0.4818, respectively).

## 4. Discussion

In this study, we evaluate, for the first time, ADMR in Brazil over the years from 2010 to 2020 and its potential correlation with factors associated with demographic transition phenomena and pesticide sales during the same period. The present study shows that LEB in Brazil had an important evolution between the years 2010 and 2020 (Figure 2), a fact that may be related with the improvement in living conditions of the Brazilian people. Indeed, LEB can be defined as the number of years of life expected for a newborn in each geographic space, if the pattern of mortality observed in the period is maintained and its increase is directly related to the improvement in the living conditions of that population. According to IBGE [19], the mortality rate per thousand inhabitants was registered in Brazil to be 5.8 in 2010 and 7.4 in 2020, with an average ± standard deviation (SD) of 6.3 ± 0.2. Considering that 2020 was atypical because of several deaths induced by the coronavirus, mortality rate was kept stable between the years 2010 (5.8) and 2019 (6.4).

Figure 2 also shows that LEB was superior in the South and Southeast regions for male, female and both sexes. The female sex presented the biggest average for LEB of 80.85 years in the South region and the male sex presented the biggest LEB of 74.07 years in the South region. The gain in LEB observed for women was 2.9 years and for men it was 3.1 years in the period from 2010 to 2020. The different patterns of morbidity and mortality in group populations are determined by multiple aspects such as unequal income distribution, environmental exposure factors, access to health services and insufficient investment in social policies. According to a study published in 2002 [22], in the South and Southeast regions, greater exposure to risk factors for respiratory diseases, such as those related to climate, urban pollution and crowding, was observed. On the other hand, a greater coverage of basic sanitation services, greater access to health services and better nutritional conditions were observed, meaning that children were less exposed to risk factors for mortality from diarrheal diseases.

The improvement in LEB demonstrated by the present study has raised an important question about the emergence of diseases related to the aging of the population. Figure 3 illustrates ADMR in Brazilian regions between 2010 and 2020. The female sex presented the biggest ADMR in the South and Southeast without statistical difference between these two regions (*p* = 0.0817). The South region registered the highest ADMR in 2019 and the Southeast region registered the highest ADMR in 2020 for the female sex. The highest ADMR was registered for the male sex in the South region in 2019. When both sexes were considered, the highest ADMR was also registered in the South region in 2019. According to previous work published about the ADMR of the United States from 1999 to 2018, it was also found that the female group was predominant in deaths caused by AD [23].

In this context, although the exact mechanisms involved in the sexual dimorphism observed in women with AD is not well established, evidence points to the role of the sexual hormones estrogens and androgens, such as 17 β-estradiol, estrone [24] and testosterone [25], which appear to promote neuroprotective effects. However, considering the reduction in estrogen levels in more advanced ages, such as those consistent with the age group with the highest prevalence of AD, it is suggested that the reduction in the estrogenic neuroprotective effect is more preponderant than that of an androgenic effect, which may be one of the factors involved in the sexual difference in ADMR [26]. In fact, other authors found that women had a 30% higher risk of dying from AD than men [27].

Figure 4 corroborates the idea that the increase in LEB has contributed to higher ADMR in Brazil over the years from 2010 to 2020. The correlation coefficients of all regions and Brazil as a whole were higher than 0.97, which indicates a strong positive correlation between ADMR and LEB with statistical significance. LEB in Brazil over the years from 2010 to 2020 was raised continuously with a medium percentage of 3.6% of increase among the five regions for both sexes. Indeed, some authors have found significant neuroinflammation in people older than 65 years, which may explain why elderly people are more vulnerable to effects induced by AD [28].

HDI is a widely adopted indicator for measuring sustainable development in socio-economy in the world [29]. This index is based on access to knowledge, on a long and healthy life and on a decent standard of living. Figure 5 presents the accumulated HDI for the five major regions of Brazil over the years from 2010 to 2020. All regions presented a continuously increase in HDI from 2010 to 2019. This tendency was stopped in 2020, a fact that could be related to the beginning of the coronavirus pandemic, with the first case registered in the month of February 2020 in Brazil [30]. The Southeast region registered the highest HDI (0.810 in 2019) and the Northeast the lowest HDI (0.659 in 2010) with statistical significance. Some authors found that the Southeast region had 157 public healthcare facilities, which represents around 35% of the entire country (Brazil). These data highlighted the greater development of this region when compared to the others [31].

In the concept of HDI, it is possible to analyze three different aspects, such as (1) long and healthy life (which is reflected by LEB), (2) expected years of schooling (knowledge) and (3) a decent standard of life, which is measured by the income distribution index (Gini index) [7]. The association between HDI and ADMR has been of interest recently, with a few works addressing this subject. In the present study, Figure 6 shows a strong positive correlation of 0.8802 which is statistically significant (*p* = 0.0004). Another study [32] performed in Brazilian state capitals also found that the educational level and HDI factors positively correlated with AD mortality ranging from r = 0.48 to 0.59. It is important to point out that the first aspect of HDI analyzes life expectancy at birth and the results of Figure 4 contribute strongly to the high correlation number found for the variables HDI versus ADMR.

As the risk of developing AD can be increased by numerous environmental and genetic factors [33], the present work also analyzed environmental factors that may be related to the gradual increase in ADMR. Among several environmental pollutants, the buying and selling of pesticides is controlled in Brazil, in contrast to many other chemicals [34]. It is known that most pesticides exhibit properties such as the capacity to trigger amyloid beta accumulation, neuronal degeneration, oxidative stress and mitochondrial dysfunction [35]. As Brazil has become one of the greatest markets of pesticides in the world [36,37], the present work correlated pesticide sales in Brazilian regions to ADMR over the years from 2010 to 2020, and Pearson’s coefficient of all five regions (Figure 7A) was statistically significant (*p* < 0.05). Pesticide sales have increased in recent years because of increased agricultural production, and the risk of exposure to these compounds has also increased; this could become a serious problem for public health, including an increase in the incidence of neurodegenerative diseases such as AD [38]. Organophosphates and some herbicides are the classes of chemicals that could be responsible for the increased risk of developing AD [39,40].

In the present work, we did not perform an analysis of the levels of pesticides in human samples to estimate human exposure. However, according to other work [41], a higher use of pesticides in some geographic regions was considered an indication of higher potential exposure for humans. In this context, the results presented in Figure 7B corroborate the results of Figure 7A and indicate a strong positive correlation when comparing acute pesticide intoxication and ADMR in the North, Northeast and South regions, with this last region having the highest ADMR when compared to the other regions. The Southeast and Midwest were the regions that did not present a significative correlation when comparing acute pesticide intoxication and ADMR, and the explanation is related to the underreporting observed in Brazil due to the start of COVID-19 in March 2020. It is important to point out that chronic exposures at low doses are not included in the results of Figure 7B, which may indicate even more alarming numbers [13]. In addition, the nature of a degenerative disease can explain the substantial delay between cumulative exposures to pesticides and the onset of clinical signs, but the growth in the use of pesticides in Brazil has been occurring since the 1960s [42] and reducing the use of pesticides becomes essential in public policies that aim to control the increase in ADMR.

The present study discusses the increase in ADMR and the possible factors that could be related to this phenomenon. Although statistically significant differences were observed, it is known that several factors may contribute to the socio-economic variables assessed in the present study (LEB and HDI). For future works, it will be necessary to unravel the other factors involved in the HDI concept, such as the educational level and decent standard of life. Even with these limitations, it was possible to identify a strong positive correlation between socio-economic factors and ADMR. Furthermore, pesticides are not the only class of environmental pollutants that could increase the risk of developing AD [43], but any conclusion made with other types of chemicals that do not have rigorous marketing control may generate inaccurate results.

## 5. Conclusions

In conclusion, the present work shows a significant increase in LEB and ADMR over the years from 2010 to 2020 in all Brazilian regions. The female population in the South region was the most affected. Moreover, the strong positive correlation between LEB and ADMR in all Brazilian regions over the years studied highlights the need for investments in public policies, on a national scale, that aim to minimize the effects of changes in demographic transitions suffered by the elderly population in the regions of Brazil. In addition, the strong positive correlation between the quantity of pesticides sold and ADMR and the progressive number of cases of intoxications caused by pesticides in Brazil signal the need for policies that prevent the irrational use of this type of chemicals.

## Data Availability

The authors confirm that the data supporting the findings of this study are available within the article.

## Abbreviations

AD: Alzheimer’s disease; ADMR: Alzheimer’s disease mortality rate; LEB: life expectancy at birth; IBGE: Brazilian Institute of Geography and Statistics; DATASUS: Department of Informatics and Technology of the Brazilian Ministry of Health; IBAMA: Brazilian Institute of the Environment and Renewable Natural Resources; HDI: Human Development Index; Gini index: the inequality of income distribution among the population of a place.
